# How minimally interrupted direct oral anticoagulants affect intraprocedural anticoagulation during atrial fibrillation ablation? Insights from a Japanese single‐center retrospective study

**DOI:** 10.1002/joa3.12228

**Published:** 2019-08-16

**Authors:** Masahiro Mizobuchi, Atsushi Funatsu, Tomoko Kobayashi, Shigeru Nakamura

**Affiliations:** ^1^ Cardiovascular Center Kyoto Katsura Hospital Kyoto Japan

**Keywords:** activated clotting time, atrial fibrillation, catheter ablation, direct oral anticoagulant

## Abstract

**Background:**

Data are still lacking regarding the effects of minimally interrupted direct oral anticoagulants (MID) on the intensity of intraprocedural anticoagulation of atrial fibrillation (AF) ablation.

**Methods:**

A total of consecutive 269 patients who undergone AF ablation were eligible for the study. All oral anticoagulants (OACs) were discontinued just one dose before the procedure except warfarin. We assessed the total required dose of UFH and time‐to‐target ACT > 300 seconds (TTA) for each of direct oral anticoagulant (DOAC) groups compared with the uninterrupted warfarin group.

**Results:**

DOACs were used in 86% of the patients in the present study (dabigatran group (DG)‐17%, rivaroxaban group (RG)‐30%, apixaban group (AG)‐29%, and edoxaban group (EG)‐10%). DG and EG used comparable dose of total UFH to WG (WG vs DG; 206 ± 53 U/kg vs 231 ± 63 U/kg; *P* = .664, vs EG; 239 ± 67 U/kg; *P* = .335), while RG and AG required higher total UFH (WG vs RG; 206 ± 53 U/kg vs 270 ± 63 U/kg; *P* < .001, vs AG; 263 ± 62 U/kg; *P* < .001). TTA was significantly longer in RG (RG:73 ± 28 minutes vs WG:51 ± 25 minutes; *P* = .001), AG (AG:64 ± 26 minutes vs WG:51 ± 25 minutes; *P* = .02), and EG (EG:67 ± 34 minutes vs WG:51 ± 25 minutes; *P* = .02) than WG, whereas DG was comparable to WG (DG:51 ± 29 minutes vs WG:51 ± 25 minutes; *P* = NS). Especially, only RG demonstrated significantly slower increase in ACT than WG (*P* = .013). In the multivariate analysis, warfarin or dabigatran use, age > 75 years, and body weight < 60 kg are clinical predictors for achieving TTA within 60 minutes (TTA‐60).

**Conclusion:**

MID‐dabigatran was comparable to uninterrupted warfarin, whereas MID‐factor Xa inhibitors were not. MID is a feasible protocol; however, we should be aware of its effect on the intraprocedural anticoagulation and differences among DOACs in the responsiveness to heparin.

## 1 INTRODUCTION

Atrial fibrillation (AF) is a critical risk factor for cerebral thromboembolism and mortality.^1^ While the Atrial Fibrillation Follow‐Up Investigation of Rhythm Management (AFFIRM) study^2^ failed to demonstrate the superiority of rhythm control over rate control in terms of mortality, several studies after the AFFIRM trial demonstrated that rhythm control with AF ablation led to improvements in quality of life,^3^ exercise tolerance,^4^ and mortality.^5^ Specifically, catheter ablation for AF is more effective in maintaining sinus rhythm than antiarrhythmic drug therapy alone. Current consensus and guidelines^6^, ^7^ recommend the consideration of AF ablation as a first‐line therapy in patients with symptomatic AF.

One of the important issues in AF ablation is periprocedural stroke. For example, in 2006, periprocedural stroke was noted in 1.1% of patients.^8^ However, the risk of stroke reduced to 0.098%.^9^ The improvement in thromboembolic complications has been owing to several factors, including continuation with vitamin K antagonists (VKAs) as a periprocedural anticoagulation protocol,^10^ monitoring of activated clotting time (ACT) during the procedure,^11^ and incorporation of preprocedural transesophageal echocardiography and irrigation catheter ablation system.

The feasibility and safety of uninterrupted direct oral anticoagulant (OAC) treatment for AF ablation are currently investigated. Cappato et al reported the first randomized trial (VENTURE‐AF) comparing uninterrupted rivaroxaban with uninterrupted VKA therapy for catheter ablation of AF^12^ and demonstrated that uninterrupted rivaroxaban was feasible and that adverse events were comparable to those observed with uninterrupted VKA therapy. Calkins et al demonstrated the superiority of uninterrupted dabigatran to warfarin regarding major bleeding events in the RE‐CIRCUIT trial.^13^ Continuous apixaban and edoxaban therapies were also comparable to uninterrupted warfarin in the AXAFA‐AFNET 5 and ELIMINATE‐AF trials.^14^, ^15^ Although these studies proved the noninferiority or the superiority of uninterrupted direct OACs compared with uninterrupted VKA therapy in terms of feasibility and safety, concerns remain regarding serious bleeding complications because antidotes for factor Xa inhibitors are not still readily available in many countries including Japan.

Minimally interrupted direct OAC (MID) is a proposed alternative periprocedural protocol for AF ablation that is expected to reduce the risk of major bleeding complications.^16^ The MID protocol is based on skipping one or two doses of OACs before ablation. Although MID is assumed to be a safe oral anticoagulation protocol for reducing major bleeding complications and to be equivalent to uninterrupted VKA therapy for its capability of effective intraprocedural anticoagulation, real‐world data on the feasibility of MID in daily clinical practice of AF ablation remain limited. Thus, the aim of the present study was to elucidate the effects of MID on intraprocedural anticoagulation during AF ablation and to demonstrate the feasibility and safety of MID in periprocedural OAC management for AF ablation. In the current study, serial changes in ACT and time‐to target ACT (TTA) were used as surrogate markers to assess the suitability of intraprocedural anticoagulation using different direct OACs, including dabigatran, rivaroxaban, apixaban, and edoxaban, in comparison with uninterrupted VKA therapy.

## 2 METHODS

### 2.1 Study design and population

This was a retrospective, single‐center, observational study. The study protocol adhered to the Declaration of Helsinki and was approved by the Ethics Committee of Kyoto Katsura Hospital (No.648). A total of 272 consecutive patients with nonvalvular AF who underwent AF ablation at Kyoto Katsura Hospital between November 2014 and March 2018 were eligible to be included in the present study. OACs were unchanged for at least three weeks before and 30 days after the procedure during the perioperative period. Patients who received under or overdoses of OACs were instructed to adjust or correct to their appropriate doses based on their renal function, body weight, and age during their hospital stay for ablation.

### 2.2 Periprocedural oral anticoagulation management

All OACs, whether VKAs or direct OACs, were discontinued except for a single morning dose on the day before the ablation; therefore, there was a minimum of 24 hours between the last OAC dose and ablation, including the once daily direct OACs rivaroxaban and edoxaban. To ensure compliance with the protocol during the hospital stay for AF ablation, OACs were withheld or otherwise administered under the observation by nursing stuff. VKA therapy was continued only if prothrombin time (PT)‐international normalized ratio (PT‐INR) was below the therapeutic range, which was 2.0‐3.0 for patients younger than 75 years of age and >1.6 for those over 75 years of age old according to the current guidelines of the Japanese Circulation Society.^17^ Patients who skipped more than two doses of OACs before the procedure were excluded from the analysis.

### 2.3 Anticoagulation during the procedure

Unfractionated heparin (UFH) was used for intraprocedural anticoagulation. Intravenous UFH was administered at 100 U/kg as an initial bolus immediately after the initial transseptal puncture. ACT was measured every 20 minutes after the initial UFH administration, and additional UFH was administered at 50‐60 U/kg every 20 minutes to achieve and maintain an ACT of >300 seconds based on the measured ACT.

### 2.4 AF ablation

Transesophageal echocardiography (TEE) was performed in all patients within one week before the ablation to exclude left atrial thrombus and valvular heart diseases. Transthoracic echocardiography (TTE) was required within 1 month before the ablation to evaluate cardiac function and the next day after the procedure to assess for cardiac complications due to the ablation. The following TTE parameters before ablation were used for baseline: left atrial diameter, left atrial volume index, and left ventricular ejection fraction. TTE the day after the ablation was allowed if no TTE was performed before the ablation. Plasma coagulation markers including PT and activated partial thromboplastin time were measured before each session.

Venous access was established through the right or left femoral vein and the right internal jugular vein. A 8.5‐F Fast‐Cath™ Swartz™ introducer sheath and/or Agilis™ NxT steerable introducer (St. Jude Medical Inc.) were inserted via femoral veins to place the intracardiac echocardiography catheter (Ultra ICE™ Plus, Boston Scientific Corporation), the circular mapping catheter, and the ablation catheter. Transseptal access was established with the NRG™ Transseptal Needle (Baylis Medical Company, Inc.). A duodecapolar electrode catheter for coronary sinus was also inserted through a 6‐F sheath from the right internal jugular vein (BeeAT™, Japan Lifeline Co., Ltd). A size‐adjustable duodecapolar circular mapping catheter (Inquiry Optima™ PLUS, St. Jude Medical) was placed in the ostium of each pulmonary vein (PV) to record electrical activity. Left atrium (LA) and PVs were reconstructed with a three‐dimensional (3D) electro‐anatomical mapping system (EnSite Velocity™, St. Jude Medical). Circumferential PV isolation was performed with a 4‐mm‐tip saline‐irrigated radiofrequency ablation catheter (Flexability™, St. Jude Medical). Radiofrequency energy was delivered at 25‐35 W and an 8‐13 mL/min flow rate with a maximal temperature of 42°C. Cryoballoon ablation was performed with Arctic Front Advance™ (Medtronic Inc, Minneapolis, MN) and an Achieve™ 8‐pole mapping catheter (Medtronic), which were inserted via a 12‐F steerable sheath (FlexCath™ advance steerable sheath, Medtronic). Cryoenergy was applied for 180‐240 seconds to each PV. After successful PV isolation, isoproterenol (10‐20 μg/mL) was administered. At least 30 minutes after successful PV isolation, electrical activity in each PV was reconfirmed by pacing on the inside and the antrum of each PV to confirm entrance/exit block and by intravenous adenosine triphosphate to unveil dormant conductions. If AF remained after successful PV isolation, intracardiac cardioversion (10‐30 J) was performed to restore sinus rhythm. In patients with nonparoxysmal AF, additional linear ablations were performed for the LA roof, bottom, and mitral isthmus. If macroreentrant atrial tachycardias (AT) were inducible with atrial burst pacing from the coronary sinus electrodes, additional RF ablations were performed to eliminate inducible AT. All ablation procedures were performed under conscious or deep sedation with an initial bolus of pentazocine and hydroxyzine pamoate and continuous dexmedetomidine administration. After completion of the ablation, protamine (30 mg) was administered to reverse the effect of heparin, and all sheaths were removed. All patients were followed at 1 and 6 months after the ablation.

### 2.5 Statistical analysis

All continuous variables were expressed as means ± standard deviation or medians with interquartile ranges. All categorical variables were reported as number (percentage) of patients. Unpaired Student's *t* test and one‐way analysis of variance (ANOVA) were used to compare continuous variables. Serial changes in ACT were evaluated by repeated‐measures ANOVA. Categorical variables were compared with the chi‐squared test or Fisher's exact test. A *P*‐value of <.05 was considered statistically significant. Logistic regression analysis was performed for the evaluation of clinical predictors of TTA within 60 minutes (TTA‐60). All results were analyzed with SPSS base 11.0J for Windows.

## 3 RESULTS

### 3.1 Patient population and OAC distribution

All patients were followed at 1 and 6 months. Of a total of 272 patients who underwent AF ablation during the study period, three patients were excluded because of the lack of preprocedural OACs due to hemodialysis (n = 2) and poor adherence (n = 1). Consequently, a total of 269 patients were included in the final analyses. The baseline characteristics of the study cohort are presented in Table 1. Warfarin was used in 14% of the patients, whereas direct OACs were used in 86% of the patients and included dabigatran (17%), rivaroxaban (30%), apixaban (29%), and edoxaban (10%). The mean age was significantly higher in the apixaban and edoxaban groups compared with the other groups. There was a difference in sex distribution, especially in the dabigatran group. Inappropriate OAC doses were observed in the rivaroxaban (9.9%; overdose/underdose, 2/6), apixaban (10%; overdose/underdose, 2/6), and edoxaban (15%; overdose/underdose, 4/0) groups. The body weight was significantly lower in the apixaban group. No significant differences in CHADS_2_ scores or cardiac function on TTE were observed among the groups. The treatment interruption was significantly longer for the OACs with once daily dosing (warfarin, rivaroxaban, and edoxaban) than those with twice daily dosing (dabigatran and apixaban) (mean treatment interruption, 25.3 ± 3.4 and 16.9 ± 2.2 hours for once daily and twice daily dosing, respectively; *P* < .0001). The details related to interruption durations are shown in Table 1.

**Table 1:** Patient characteristics

	Warfarin (WG; n = 37)	Dabigatran (DG; n = 45)	Rivaroxaban (RG; n = 81)	Apixaban (AG; n = 79)	Edoxaban (EG; n = 27)	*P*
Age (years)	65 ± 8.8	65 ± 9.4	65 ± 12	71 ± 8.0	68 ± 13	.003
Sex (Male/female)	26/11	41/4	56/25	46/33	19/8	.005
Body weight (kg)	68 ± 13	66 ± 14	64 ± 11	60 ± 12	69 ± 13	.005
Serum Cr (mg/dL)	1.19 ± 2.05	0.85 ± 0.15	0.80 ± 0.18	0.84 ± 0.20	0.94 ± 0.33	.135
CHADS_2_ score	1.0 ± 0.9	0.9 ± 1.0	0.9 ± 0.9	1.3 ± 1.0	1.3 ± 1.0	.055
PT (sec)	23 ± 5.9	14 ± 1.2	13 ± 1.3	14 ± 1.4	13 ± 1.6	<.001
PT‐INR	1.9 ± 0.5	1.2 ± 0.1	1.1 ± 0.1	1.2 ± 0.1	1.1 ± 0.1	<.001
APTT (sec)	32 ± 4.3	36 ± 5.2	30 ± 4.1	29 ± 3.4	30 ± 3.8	<.001
LAD (mm)	42 ± 7.7	40 ± 7.1	40 ± 6.7	40 ± 5.9	44 ± 8.7	.094
LAVI (mL/m^2^)	39 ± 16	33 ± 13	35 ± 14	33 ± 11	41 ± 25	.096
LVEF (%)	66 ± 6.7	64 ± 9.6	65 ± 9.3	66 ± 8.4	63 ± 11	.574
DOAC overdose	n/a	0	2	2	4	.002
DOAC underdose	n/a	0	6	6	0	
Interruption (hours)	23.5 ± 4.8	17.0 ± 2.2	25.9 ± 2.4	16.9 ± 2.1	25.8 ± 2.3	<.001

Note

Values are mean ± SD or the number.

Abbreviations: APTT, activated partial thromboplastin time; CHADS_2_, congestive heart failure, hypertension, Age ≧ 75 years, diabetes mellitus, stroke/transient ischemic attack; Cr, creatinine; DOAC, direct oral anticoagulant; LAD, left atrial diameter; LAVI, left atrial volume index; LVEF, left ventricular ejection fraction; PT, prothrombin time; PT‐INR, international normalized ratio of prothrombin time.

### 3.2 UFH and mean ACT

There were significant differences in mean initial and additional UFH doses among the OACs, although the initial and mean additional UFH doses were predetermined at 100 and 50‐60 U/kg, respectively. Compared with the warfarin group (98 ± 12 U/kg), the rivaroxaban (103 ± 12 U/kg, *P* = .035) and apixaban (106 ± 13 U/kg, *P* = .001) groups required higher doses for the initial UFH dose. Similarly, the rivaroxaban (58 ± 13 U/kg, *P* = .023) and apixaban (58 ± 18 U/kg, *P* = .022) groups also required significantly higher mean additional bolus UFH doses compared with the warfarin group (50 ± 19 U/kg). The dabigatran (231 ± 63 U/kg, *P* = .664) and edoxaban (239 ± 67 U/kg, *P* = .335) groups required total UFH doses that were comparable with that required in the warfarin group (206 ± 53 U/kg), whereas the rivaroxaban (270 ± 63 U/kg, *P* < .001) and apixaban (263 ± 62 U/kg, *P* < .001) groups required significantly higher total UFH doses compared with the warfarin group.

All factor Xa inhibitors (305 ± 29, 284 ± 23, and 282 ± 33 seconds for rivaroxaban, apixaban, and edoxaban; *P* < .001, *P* < .001, and *P* = .005, respectively) demonstrated lower mean ACT values compared with warfarin (283 ± 25 seconds). Conversely, the mean ACT value of the dabigatran group (305 ± 27 seconds, *P* = .942) was comparable to that of the warfarin group (Table 2).

**Table 2:** Heparin dose, mean ACT and mean time‐to‐target ACT (TTA)

	Warfarin (WG; n = 37)	Dabigatran (DG; n = 45)	Rivaroxaban (RG; n = 81)	Apixaban (AG; n = 79)	Edoxaban (EG; n = 27)	*P*
Initial bolus UFH (U/kg)	98 ± 12	103 ± 7.6	103 ± 12	106 ± 13	105 ± 3.7	.02
Additional UFH (U/kg)	50 ± 19	46 ± 23	58 ± 13	58 ± 18	53 ± 14	.001
Total UFH (U/kg)	206 ± 53	231 ± 63	270 ± 63	263 ± 62	239 ± 67	<.001
Mean ACT (sec)	305 ± 29	305 ± 27	283 ± 25	284 ± 23	282 ± 33	<.001
Time‐to‐target ACT (min.)	51 ± 25	51 ± 29	73 ± 28*	64 ± 26§	67 ± 39¶	<.001

Note

Values are mean ± SD.

Abbreviations: ACT, activated clotting time; UFH, unfractionated heparin.

* *P* < .001.

^§^ *P* = .022.

^¶^ *P* = .023.

### 3.3 Mean TTA and transition of the change in ACT

As shown in Table 2, the mean TTA was comparable between the dabigatran and warfarin groups. Factor Xa inhibitors (rivaroxaban, apixaban, and edoxaban) demonstrated significantly longer TTA than warfarin.

**Figure 1:**
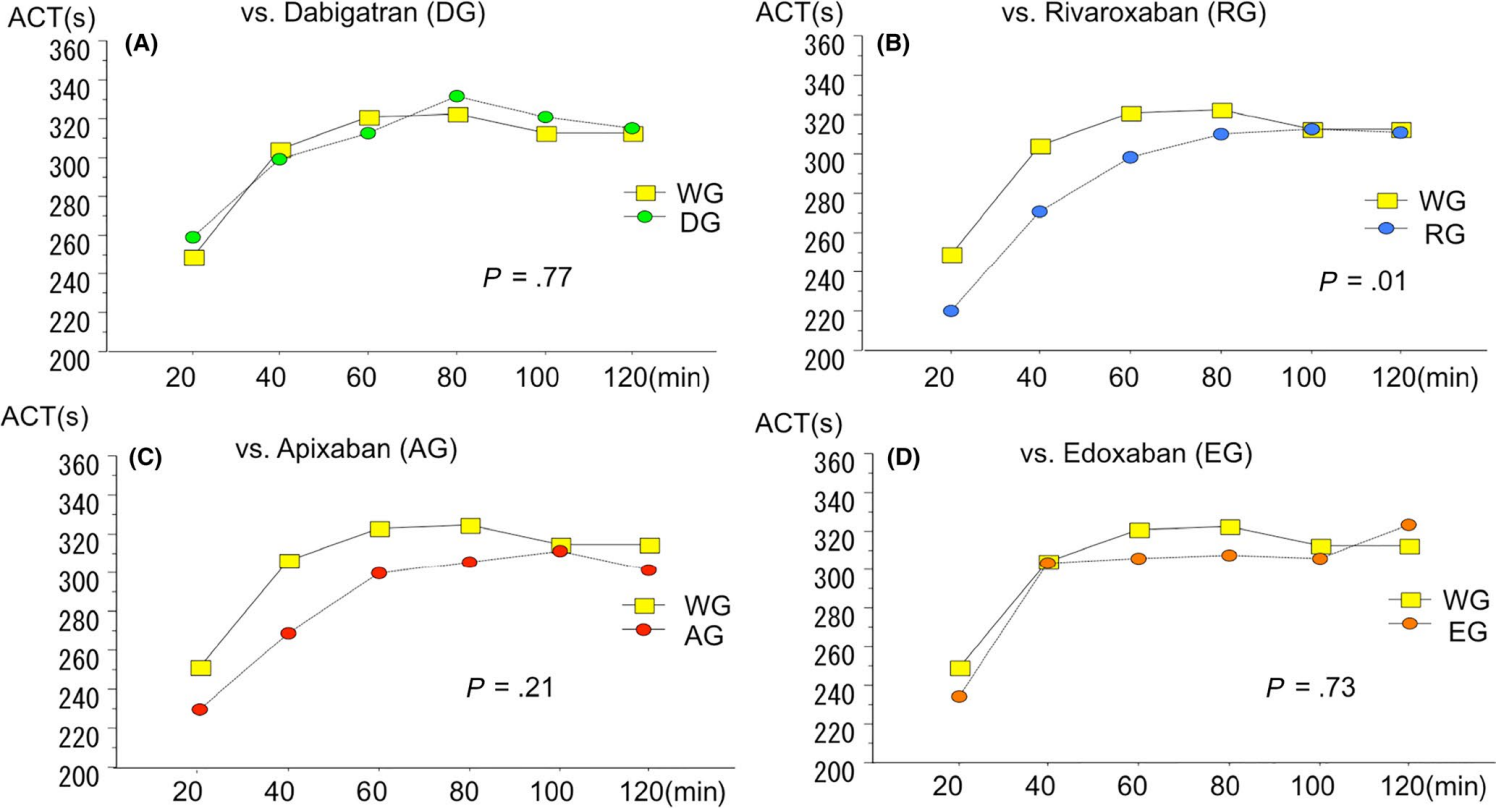
Changes in activated clotting time during atrial fibrillation ablation. Horizontal lines represent time after initial heparin bolus administration during atrial fibrillation ablation (min). Vertical lines represent activated clotting time (ACT) values (s). Graphs depict the comparison of ACT transitions during ablation between the warfarin group (WG) and the direct oral anticoagulant groups. (A) WG vs dabigatran group (DG) [*P* = .77, repeated‐measures analysis of variance (ANOVA)]. (B) WG vs rivaroxaban group (RG) (*P* = .013, repeated‐measures ANOVA). (C) WG vs apixaban group (*P* = .21, repeated‐measures ANOVA). (D) WG vs edoxaban group (EG) (*P* = .73, repeated‐measures ANOVA)

The time course of ACT values measured overtime for each direct OAC is shown in Figure 1. Compared with the warfarin group, there were no statistically significant differences in the dabigatran (*P* = .77; Figure 1A), apixaban (*P* = .21; Figure 1C), and edoxaban (*P* = .73; Figure 1D) groups. However, only the rivaroxaban group demonstrated a late catch‐up type change in ACT and a significantly slower increase in ACT compared with the warfarin group (*P* = .013, Figure 1B).

### 3.4 Clinical predictors for TTA‐60

Female sex, body weight of ≤60 kg, age of ≥75 years, and warfarin and dabigatran use were more frequent in the TTA‐60 group, whereas rivaroxaban use in the non‐TTA‐60 group was significantly more frequent than that in the TTA‐60 group (Table 3). Based on these results, the clinical predictors for TTA‐60 were evaluated by multivariate analysis. As shown in Table 4, body weight of <60 kg, age of >75 years, and warfarin and dabigatran use were clinical predictors for TTA‐60. Conversely, rivaroxaban use was a risk factor for non‐TTA‐60.

**Table 3:** Univariate analysis of patient backgrounds for TTA‐60

	TTA60 (N = 185)	Non‐TTA60 (N = 84)	*P*
Female sex	63 (78)	18 (22)	.044
Body weight ≦60 kg	82 (44)	17 (20)	<.001
CHADS_2_ score ≧ 2	51 (28)	25 (30)	.771
Age ≧ 75	57 (31)	12 (14)	.004
Serum Cr (mg/dL)	0.9 ± 0.9	0.8 ± 0.2	.488
Warfarin‐use	31 (84)	6 (16)	.036
Dabigatran‐use	38 (84)	7 (16)	.013
Rivaroxaban‐use	41 (51)	40 (49)	<.001
Apixaban‐use	25 (60)	17 (40)	.563
Edoxaban‐use	19 (70)	8 (30)	>.999
LAD (mm)	41 ± 6.9	42 ± 7.3	.287
LAVI (mL/m^2^)	35 ± 14	35 ± 17	.917
LVEF (%)	65 ± 9.1	65 ± 8.8	.544

Note

Values are mean ± SD or the number(%). TTA‐60, time‐to‐target ACT within 60 minutes.

Abbreviations are shown in Table 1.

**Table 4:** Multivariate analysis of clinical predictors for achieving TTA‐60

	Multivariate OR (95%CI)	*P*
Female sex	1.380 (0.678‐2.806)	.374
Body weight ≦ 60kg	2.797 (1.396‐5.603)	.004
CHADS_2_ score ≧ 2	0.752 (0.391‐1.449)	.395
Age ≧ 75 years	2.903 (1.346‐6.260)	.007
Warfarin‐use	3.109 (1.134‐8.529)	.028
Dabigatran‐use	3.199 (1.224‐8.363)	.018
Rivaroxaban‐use	0.478 (0.250‐0.912)	.025

OR, odds ratio (95% of confidence interval; CI).

### 3.5 Complications

Pericardial effusion (PE), including hemopericardium, occurred in five patients, with an incidence of 1.86%. Rivaroxaban and warfarin were used in two and three patients, respectively. The characteristics of these patients are presented in Table 5. The rate of warfarin use and body weight were significantly higher in the PE group than the non‐PE group, whereas there were no significant differences in any of the other baseline characteristics between the two groups. One patient with PE required surgical repair, whereas the remaining patients recovered by pericardiocentesis and anticoagulation reversal with protamine.

**Table 5:** Comparison of characteristics of patients with pericardial effusion

	PE(+) (n = 5)	PE(−) (n = 264)	*P*
Age (years)	58 ± 14	67 ± 10	.068
Male sex	4 (80)	184 (70)	.99
Body weight (kg)	80 ± 9.0	64 ± 12	.004
Serum Cr (mg/dL)	0.75 ± 0.11	0.88 ± 0.76	.697
CHADS_2_ score	0.8 ± 0.4	1.0 ± 1.0	.554
Persistent AF	0 (0)	71 (27)	.33
PTINR	1.54 ± 0.55	1.25 ± 0.36	.073
APTT (sec)	32 ± 4.0	31 ± 4.9	.497
Warfarin‐use	3 (60)	34 (13)	.020

Values are mean ± SD or the number(%). Abbreviations are shown in Table 1.

**Figure 2:**
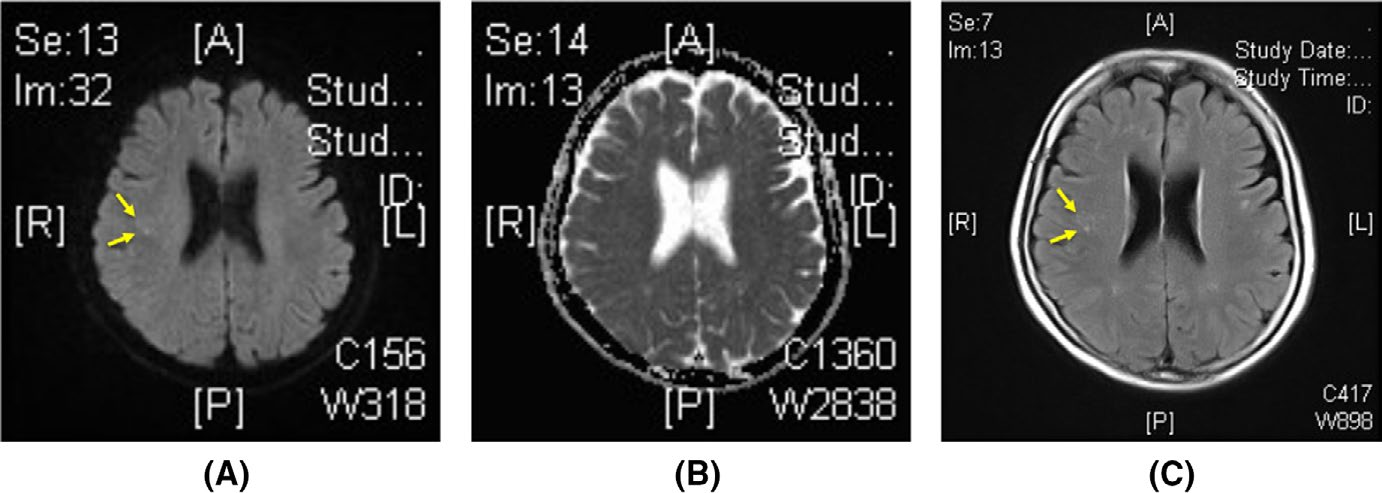
Brain magnetic resonance images (MRI) of a patient who underwent AF ablation and developed subacute silent stroke. (A) Diffusion‐weighted image (DWI), (B) Apparent diffusion coefficient (ADC) map, and (C) fluid‐attenuated inversion recovery (FLAIR) image. The patient complained of mild dizziness 3 weeks after the AF ablation. The brain MRI images reveal a round‐shaped lesion (arrows) in the right parietal lobe suggesting subacute stroke. No abnormal findings are observed in cerebellum or brain stem

Thromboembolism within 30 days was recognized in one patient in the rivaroxaban group (0.37%). The female patient complained of mild dizziness 3 weeks after the procedure. Brain magnetic resonance imaging revealed a subacute stroke in the right parietal lobe that was not related to her symptoms (Figure 2). The patient recovered shortly without any residual neurological deficits.

## 4 DISCUSSION

Accumulating evidence and advances in technologies and skills established AF ablation as a cornerstone therapeutic approach. Currently, direct OACs play a central role in anticoagulation therapy for AF by replacing VKAs due to their noninferiority/superiority in the prevention of ischemic stroke and the lower risk of bleeding compared with VKAs.^18^, ^19^, ^20^, ^21^, ^22^ However, evidence is limited regarding the outcomes of concomitantly used intraprocedural heparin and direct OACs during AF ablation. Of note, Asians are at a higher risk of intracranial hemorrhage than non‐Asians during treatment with VKAs.^23^ Furthermore, AF ablation is currently indicated even for elderly patients who are prone to bleeding.^24^ In the era of AF ablation, the safety of periprocedural direct OAC dosing should be elucidated.

Several studies demonstrated the characteristics of MID for AF ablation by comparing with uninterrupted warfarin^25^, ^26^, ^27^ and reported that uninterrupted warfarin consistently demonstrated shorter TTA values than MID. However, the study design in these trials was advantageous for uninterrupted warfarin. Konduru et al,^25^ Bassiouny et al,^26^ and Armbruster et al^27^ compared uninterrupted warfarin with 1‐3 doses of interrupted direct OACs. Since the half‐life of direct OACs is generally within 12 hours,^28^ skipping more than two doses are sufficient for negating their anticoagulant effects. Consequently, these studies indeed compared the effects of uninterrupted warfarin (PT‐INR > 2.0) with DOACs of underdose. Thus, it is not fair to conclude that the MID was inferior to uninterrupted warfarin for intraprocedural anticoagulation based on results of these previous studies. In the present study, rivaroxaban and apixaban required significantly higher doses of UFH compared with warfarin throughout the procedure, whereas dabigatran and edoxaban did not. Conversely, previous studies reported that dabigatran required higher heparin doses.^26^, ^29^ The disagreement between the current and the two previous studies might result from differences in periprocedural PT‐INR for warfarin users and the protocol for additional UFH administration during the procedure. Most of the current patients had lower PT‐INR (1.9 ± 0.5) compared with those in the two previous studies in accordance with the guidelines of the Japanese Circulation Society.^17^ It would be attributed to diminish the advantage of warfarin against direct OACs. Furthermore, additional UFH was strictly administered in accordance with the predefined protocol, which might have contributed to the distinct responsiveness of the patients on OACs to UFH.

In the present study, we also elucidated the differences in TTA among direct OACs. Minimally interrupted dabigatran was comparable to uninterrupted warfarin, whereas minimally interrupted factor Xa inhibitors demonstrated longer TTA values. In addition, there were no significant differences in the ACT transition pattern among the warfarin, dabigatran, apixaban, and edoxaban groups; however, the rivaroxaban group demonstrated significantly slower ACT increases than the warfarin group (Figure 1). There are at least two potential explanations for the clear differences in the responsiveness to UFH. First, as long as dabigatran remains in the blood, the direct inhibition of thrombin and the indirect inhibition of the thrombin burst via the intrinsic pathway,^30^, ^31^ that is, residual inhibition of the “positive feedback” on the intrinsic pathway by thrombin itself, would be maintained. In fact, activated partial thromboplastin time was still prolonged before the ablation in the dabigatran group. Thus, the anticoagulant effect of minimally interrupted dabigatran was considered to persist even on the day of the ablation. Second, the clear difference in the responsiveness of the rivaroxaban to UFH can be partly explained by its half‐lives and daily dosing pattern in clinical settings. The half‐life of rivaroxaban is 5‐9 hours.^28^ The time between the last administration of rivaroxaban and the initial heparin bolus was likely longer than 24 hours because most patients took rivaroxaban in the morning, which should provide enough time for its anticoagulant effect to weaken or disappear before the ablation. A cultural difference might be attributed to the dose scheduling as well. Japanese patients are likely to take rivaroxaban after breakfast, whereas patients in Western countries are likely to take it after the evening meal.

The current study found that minimally interrupted dabigatran was comparable to uninterrupted warfarin for intraprocedural anticoagulation based on the ACT transition pattern and TTA and required the same dose of UFH. For minimally interrupted rivaroxaban, higher dose for the initial UFH bolus was required compared with the other OACs to achieve the target ACT.

Minimally interrupted apixaban and edoxaban demonstrated similar ACT transition pattern to uninterrupted warfarin by administering relatively higher doses of UFH. For apixaban, shorter duration of interruption presumably gave rise to persistence of anticoagulation effects. For edoxaban, the longer half‐life^28^ than other factor Xa inhibitors might explain the difference in responsiveness to UFH compared with rivaroxaban. Other factors associated with earlier TTA achievement were age of ≥75 years and body weight of <60 kg. These clinical background characteristics should thus be considered in determining optimal intraprocedural anticoagulation for AF ablation in addition to the choice of OACs.

At present, debate is ongoing regarding the establishment of uninterrupted direct OAC treatment as a uniform protocol for periprocedural anticoagulation in the clinical setting because of the lack of evidence especially for patients with bleeding risk and as rescue antidotes are not readily available for all OACs. A recent prospective randomized trial investigated optimal periprocedural direct OAC strategies by comparing MID (single‐dose skipped and 24‐hour skipped direct OACs) with uninterrupted direct OACs.^32^ The study demonstrated that MID and uninterrupted direct OACs exhibited comparable efficacy and safety in an Asian population regardless of the type of the direct OAC. However, in that study, the patients were younger (mean, 58.3 ± 11.3 years of age) and had higher body weight (over 70 kg) compared with the current study cohort. Thus, the current study patients might have been less vulnerable to bleeding, albeit being an Asian cohort. In addition, as the previous study was an open label, randomized trial, there is the possibility of performance and detection biases. Consequently, the bleeding risk associated with uninterrupted direct OAC might have been underestimated. The authors implicated that MID might be optional under the circumstance of limited availability of reversal agents.

In the present study, two patients in the minimally interrupted rivaroxaban group experienced PE and were managed with pericardiocentesis alone, whereas one patient with PE in the warfarin group required surgical repair. Thus, we consider that the MID protocol might be associated with reduced collateral damage during the procedure. We also experienced a minor TE event in a patient on the MID protocol. Since minimally interrupted factor Xa inhibitor required a longer time to achieve target ACT, TE could have been prevented by increasing the initial UFH dose to more than 100 U/kg.; however, further investigation is required to determine the appropriate dose of intraprocedural UFH for the MID protocol.

Incorporating uninterrupted and interrupted protocols for direct OACs raises concerns for serious medical errors during operations in daily clinical practice. Utmost care is necessary before introducing uninterrupted direct OAC protocols in patients undergoing AF ablation based solely on the results of several commercially funded trials.^12^, ^13^, ^14^ In fact, Ha et al reported that MID was comparable to uninterrupted VKA therapy in terms of safety and that uninterrupted direct OAC was not superiority to MID based on their meta‐analysis.^33^

We believe that MID should be considered as an alternative periprocedural direct OAC dosing protocol, especially for patients with higher bleeding risk; however, differences in responsiveness to UFH should be taken into account for optimal intraprocedural anticoagulation.

## 5 LIMITATIONS

We acknowledge several limitations in the current study. First, this was a retrospective observational study performed at a single‐center in Japan. Oral anticoagulation was performed in accordance with the guidelines of the Japanese Circulation Society. Thus, the practices were different from those in European, American, and Asian countries. The patients were not blinded, randomized, or adjusted based on their background characteristics. Second, the patient enrollment was consecutive; however, three patients not on OACs were excluded. Third, the anticoagulant effects of the direct OACs could not be accurately estimated by ACT. We recognize that ACT is merely a surrogate marker for intraprocedural anticoagulation. Fourth, we could not determine that the MID protocol and TTA‐60 guaranteed the prevention of future clinical thromboembolic or bleeding events. Fifth, we recognize underdoses of factor Xa inhibitors may confound the results, though they were not frequent (7.5% in RG, and 7.6% in AG). Furthermore, all patients were Japanese, who were fundamentally at a lower risk for TE (mean CHADS_2_, 1.1 ± 1.0). Therefore, it is prudent to confirm these results in other populations. A randomized, prospective, multicenter study to validate the safety of the MID protocol for AF ablation is required.

## 6 CONCLUSIONS

MID is a feasible option for periprocedural anticoagulation strategy for AF ablation. However, differences in responsiveness to UFH among different direct OACs should be taken into account to achieve optimal intraprocedural anticoagulation. The present study elucidated a clear difference between dabigatran, a direct thrombin inhibitor, and other factor Xa inhibitors.

## DISCLOSURE

Masahiro Mizobuchi had received speakers’ honoraria for lecturing from Boehringer Ingelheim Japan, Inc., Bayer Yakuhin, Ltd, Bristol‐Myers Squibb Company, Ltd, and Daiichi Sankyo Company, Ltd.

## CONFLICT OF INTERESTS

All authors declare no conflict of interests for this article.

## References

[1] Camm AJ , Kirchhof P , Lip G , Schotten U , Savelieva I , Ernst S , et al. Guidelines for the management of atrial fibrillation: the task force for the management of atrial fibrillation of the European Society of Cardiology (ESC). Eur Heart J. 2010;31:2369–429. 20802247 10.1093/eurheartj/ehq278

[2] Wyse DG , Waldo AL , Di Marco JP , Domanski MJ , Rosenberg Y , Schron EB , et al. A comparison of rate control and rhythm control in patients with atrial fibrillation. N Engl J Med. 2002;347:1825–33. 12466506 10.1056/NEJMoa021328

[3] Pappone C , Rosanio S , Augello G , Gallus G , Vicedomini G , Mazzone P , et al. Mortality, morbidity, and quality of life after circumferential pulmonary vein ablation for atrial fibrillation: outcomes from a controlled nonrandomized long‐term study. J Am Coll Cardiol. 2003;42:185–97. 12875749 10.1016/s0735-1097(03)00577-1

[4] Yagishita A , Yamauchi Y , Sato H , Yamashita S , Hirao T , Miyamoto T , et al. Improvement in the quality of life and exercise performance in relation to the plasma b‐type natriuretic peptide level after catheter ablation in patients with asymptomatic persistent atrial fibrillation. Circ J. 2017;81:444–9. 28123151 10.1253/circj.CJ-16-1123

[5] Barra S , Baran J , Narayanan K , Boveda S , Fynn S , Heck P , et al. Association of catheter ablation for atrial fibrillation with mortality and stroke: a systematic review and meta‐analysis. Int J Cardiol. 2018;266:136–42. 29887429 10.1016/j.ijcard.2018.03.068

[6] January CT , Wann LS , Alpert JS , Calkins H , Cigarroa JE , Cleveland JC , et al. 2014 AHA/ACC/ HRS guideline for the management of patients with atrial fibrillation: executive summary: a report of the American College of Cardiology/American Heart Association Task Force on Practice Guidelines and the Heart Rhythm Society. Circulation. 2014;130:2071–104. 24682348 10.1161/CIR.0000000000000040

[7] Calkins H , Hindricks G , Cappato R , Kim YH , Saad EB , Aguinaga L , et al. 2017 HRS/EHRA/ECAS/APHRS/SOLAECE expert consensus statement on catheter and surgical ablation of atrial fibrillation: executive summary. Heart Rhythm. 2017;14:e445–e494. 10.1016/j.hrthm.2017.07.009 31631881

[8] Oral H , Chugh A , Özaydın M , Good E , Fortino J , Sankaran S , et al. Risk of thromboembolic events after percutaneous left atrial radiofrequency ablation of atrial fibrillation. Circulation. 2006;114:759–65. 16908760 10.1161/CIRCULATIONAHA.106.641225

[9] Hussein AA , Martin DO , Saliba W , Patel D , Karim S , Batal O , et al. Radiofrequency ablation of atrial fibrillation under therapeutic international normalized ratio: a safe and efficacious periprocedural anticoagulation strategy. Heart Rhythm. 2009;6:1425–9. 19968920 10.1016/j.hrthm.2009.07.007

[10] Di Biase L , Burkhardt JD , Santangeli P , Mohanty P , Sanchez JE , Horton R , et al. Periprocedural stroke and bleeding complications in patients undergoing catheter ablation of atrial fibrillation with different anticoagulation management: results from the Role of Coumadin in Preventing Thromboembolism in Atrial Fibrillation (AF) Patients Undergoing Catheter Ablation (COMPARE) randomized trial. Circulation. 2014;129:2638–44. 24744272 10.1161/CIRCULATIONAHA.113.006426

[11] Gaita F , Caponi D , Pianelli M , Scaglione M , Toso E , Cesarani F , et al. Radiofrequency catheter ablation of atrial fibrillation: a cause of silent thromboembolism? Magnetic resonance imaging assessment of cerebral thromboembolism in patients undergoing ablation of atrial fibrillation. Circulation. 2010;122:1667–73. 20937975 10.1161/CIRCULATIONAHA.110.937953

[12] Cappato R , Marchlinski FE , Hohnloser SH , Naccarelli GV , Xiang J , Wilber DJ , et al. Uninterrupted rivaroxaban vs uninterrupted vitamin K antagonists for catheter ablation in non‐valvular atrial fibrillation. Eur Heart J. 2015;36:1805–11. 25975659 10.1093/eurheartj/ehv177 PMC4508487

[13] Calkins H , Willems S , Gerstenfeld EP , Verma A , Schilling R , Hohnloser SH , et al. uninterrupted dabigatran versus warfarin for ablation in atrial fibrillation. N Engl J Med. 2017;376:1627–36. 28317415 10.1056/NEJMoa1701005

[14] Kirchhof P , Haeusler KG , Blank B , De Bono J , Callans D , Elvan A , et al. Apixaban in patients at risk of stroke undergoing atrial fibrillation ablation. Eur Heart J. 2018;39:2942–55. 29579168 10.1093/eurheartj/ehy176 PMC6110196

[15] Hohnloser SH , Camm J , Cappato R , Diener H‐C , Heidbüchel H , Mont L , et al. Uninterrupted edoxaban vs vitamin K antagonists for ablation of atrial fibrillation: the ELIMINATE‐AF trial. Eur Heart J. ehz190 10.1093/eurheartj/ehz190 [Epub ahead of print]. PMC6754569 30976787

[16] Weitz JI , Healey JS , Skanes C , Verma A . Periprocedural management of new oral anticoagulants in patients undergoing atrial fibrillation ablation. Circulation. 2014;129:1688–94. 24753548 10.1161/CIRCULATIONAHA.113.005376

[17] Okumura K , Aizawa Y , Aihara N , et al.Guidelines for indications and procedural techniques of catheter ablation(JCS2012). Available from: http://www.j-circ.or.jp/guideline/pdf/JCS2012_okumura_h.pdf. Accessed November 28 2018.

[18] Connolly SJ , Ezekowitz MD , Yusuf S , Eikelboom J , Oldgren J , Parekh A , et al. Dabigatran versus Warfarin in Patients with Atrial Fibrillation. N Engl J Med. 2009;361:1139–51. 19717844 10.1056/NEJMoa0905561

[19] Patel MR , Mahaffey KW , Garg J , Pan G , Singer DE , Hacke W , et al. Rivaroxaban versus warfarin in nonvalvular atrial fibrillation. N Engl J Med. 2011;365:883–91. 21830957 10.1056/NEJMoa1009638

[20] Granger CB , Alexander JH , McMurray JJ , Lopes RD , Hylek EM , Hanna M , et al. Apixaban versus warfarin in patients with atrial fibrillation. N Engl J Med. 2011;365:981–92. 21870978 10.1056/NEJMoa1107039

[21] Giugliano RP , Ruff CT , Braunwald E , Murphy SA , Wiviott SD , Halperin JL , et al. Edoxaban versus warfarin in patients with atrial fibrillation. N Engl J Med. 2013;369:2093–104. 24251359 10.1056/NEJMoa1310907

[22] Chan YH , Kuo CT , Yeh YH , Chang SH , Wu LS , Lee HF , et al. Thromboembolic, bleeding, and mortality risks of rivaroxaban and dabigatran in Asians with nonvalvular atrial fibrillation. J Am Coll Cardiol. 2016;68:1389–401. 27659460 10.1016/j.jacc.2016.06.062

[23] Shen AY , Yao JF , Brar SS , Jorgensen MB , Chen W . Racial/ethnic differences in the risk of intracranial hemorrhage among patients with atrial fibrillation. J Am Coll Cardiol. 2007;50:309–15. 17659197 10.1016/j.jacc.2007.01.098

[24] Inoue K , Murakawa Y , Nogami A , Shoda M , Naito S , Kumagai K , et al. Current status of catheter ablation of atrial fibrillation in Japan: summary of the 4th survey of the Japanese Catheter Ablation Registry of Atrial Fibrillation (J‐CARAF). J Cardiol. 2016;68:83–8. 26386754 10.1016/j.jjcc.2015.08.011

[25] Konduru SV , Cheema AA , Jones P , Li Y , Ramza B , Wimmer AP . Differences in intraprocedural ACTs with standardized heparin dosing during catheter ablation for atrial fibrillation in patients treated with dabigatran vs patients on uninterrupted warfarin. J Interv Card Electrophysiol. 2012;35:277–84. 23015216 10.1007/s10840-012-9719-9

[26] Bassiouny M , Saliba W , Rickard J , Shao M , Sey A , Diab M , et al. Use of dabigatran for periprocedural anticoagulation in patients undergoing catheter ablation for atrial fibrillation. Circ Arrhythm Electrophysiol. 2013;6:460–6. 23553523 10.1161/CIRCEP.113.000320 PMC3688655

[27] Armbruster HL , Lindsley JP , Moranville MP , Habibi M , Khurram IM , Spragg DD , et al. Safety of novel oral anticoagulants compared with uninterrupted warfarin for catheter ablation of atrial fibrillation. Ann Pharmacother. 2015;49:278–84. 25515868 10.1177/1060028014563950

[28] DeLoughery TG . Practical aspects of the oral new anticoagulants. Am J Hematol. 2011;86:586–90. 21674571 10.1002/ajh.22021

[29] Nagao T , Inden Y , Yanagisawa S , Kato H , Ishikawa S , Okumura S , et al. Differences in activated clotting time among uninterrupted anticoagulants during the periprocedural period of atrial fibrillation ablation. Heart Rhythm. 2015;12:1972–8. 25881495 10.1016/j.hrthm.2015.04.016

[30] Jesty J , Beltrami E . Positive feedbacks of coagulation their role in threshold regulation. Arterioscler Thromb Vasc Biol. 2005;25:2463–9. 16179597 10.1161/01.ATV.0000187463.91403.b2

[31] Turpie A . Oral, direct factor Xa inhibitors in development for the prevention and treatment of thromboembolic diseases. Arterioscler Thromb Vasc Biol. 2007;27:1238–47. 17379841 10.1161/ATVBAHA.107.139402

[32] Yu HT , Shim J , Park J , Kim TH , Uhm JS , Kim JY , et al. When is it appropriate to stop non‐vitamin K antagonist oral anticoagulants before catheter ablation of atrial fibrillation? A multicentre prospective randamoized study. Eur Heart J. 2019;40:1531–37. 30590600 10.1093/eurheartj/ehy870

[33] Ha FJ , Barra S , Brown AJ , Begley DA , Grace AA , Agarwal S . Continuous and minimally‐interrupted direct oral anticoagulant are both safe compared with vitamin K antagonist for atrial fibrillation ablation: an updated meta‐analysis. Int J Cardiol. 2018;262:51–6. 29606512 10.1016/j.ijcard.2018.03.095

